# Generating an oilseed rape mutant with non-abscising floral organs using CRISPR/Cas9 technology

**DOI:** 10.1093/plphys/kiac364

**Published:** 2022-08-11

**Authors:** Jian Wu, Huimin Liu, Sichao Ren, Panpan Li, Xue Li, Li Lin, Qinfu Sun, Long Zhang, Chen Lin, Youping Wang

**Affiliations:** Key Laboratory of Plant Functional Genomics of the Ministry of Education, Yangzhou University, Yangzhou 225009, China; Key Laboratory of Plant Functional Genomics of the Ministry of Education, Yangzhou University, Yangzhou 225009, China; Key Laboratory of Plant Functional Genomics of the Ministry of Education, Yangzhou University, Yangzhou 225009, China; Key Laboratory of Plant Functional Genomics of the Ministry of Education, Yangzhou University, Yangzhou 225009, China; Key Laboratory of Plant Functional Genomics of the Ministry of Education, Yangzhou University, Yangzhou 225009, China; Key Laboratory of Plant Functional Genomics of the Ministry of Education, Yangzhou University, Yangzhou 225009, China; Key Laboratory of Plant Functional Genomics of the Ministry of Education, Yangzhou University, Yangzhou 225009, China; Key Laboratory of Plant Functional Genomics of the Ministry of Education, Yangzhou University, Yangzhou 225009, China; Key Laboratory of Plant Functional Genomics of the Ministry of Education, Yangzhou University, Yangzhou 225009, China; Key Laboratory of Plant Functional Genomics of the Ministry of Education, Yangzhou University, Yangzhou 225009, China; Jiangsu Key Laboratory of Crop Genomics and Molecular Breeding, Yangzhou University, Yangzhou 225009, China

## Abstract

Oilseed rape plants with abscission-defective ﬂoral organs acquired through genome editing show less susceptibility to *Sclerotinia sclerotiorum* infection and longer ﬂowering-period for flower tourism.

Dear Editor,

Oilseed rape (OSR, *Brassica napus*) is an important edible oil crop consumed worldwide. As an ornamental, its fascinating seasonal ﬂowers generate up to $1.57 million in flower tourism revenue per day in China (Xiao et al., 2021). Sclerotinia stem rot (SSR), caused by *Sclerotinia sclerotiorum* is a detrimental fungal disease for OSR. SSR is initiated by air-borne ascospores that germinate to form hyphae with the help of nutrients and moisture from abscised petals adhered to leaves and petioles (Supplemental Figure S1). These infected petals serve as secondary inocula invading other plant tissues (Hegedus and Rimmer, 2005). Petal infection is thus crucial to the prevalence of SSR in OSR. OSR varieties with abscission-defective ﬂoral organs were predicted to be less susceptible to *Sclerotinia* infection and to have a longer ﬂowering period to enhance tourism income. To date, such germplasm is unavailable in OSR. Here, we generated OSR lacking abscission of ﬂoral organs.

In Arabidopsis (*Arabidopsis thaliana*), *Inflorescence Deficient in Abscission* (*IDA*) encodes a small secreted protein with an N-terminal signal peptide, and *IDA* knockouts retain floral organs indefinitely (Butenko et al., 2003; Cho et al., 2008). Five OSR homologs to *AtIDA*, gleaned from the released OSR genome (Chalhoub et al., 2014), were cloned from the pure OSR line J9712 (Figure 1A). *BnC06.IDA* and *BnA07.IDA* share a highly conserved amino-acid sequence and exhibit 76.9% sequence similarity to *AtIDA* (Figure 1A). Transcriptomic analysis revealed that *BnC06.IDA* and *BnA07.IDA* were preferentially expressed in the floral abscission zone (AZ), and in other floral organs, including petals, sepals, and filaments. In contrast, the other three copies of *BnIDA* were barely expressed in floral organs (Figure 1B). Our results suggest a role for *BnC06.IDA* and *BnA07.IDA* in determining the fate of floral organ abscission.

**Figure 1:**
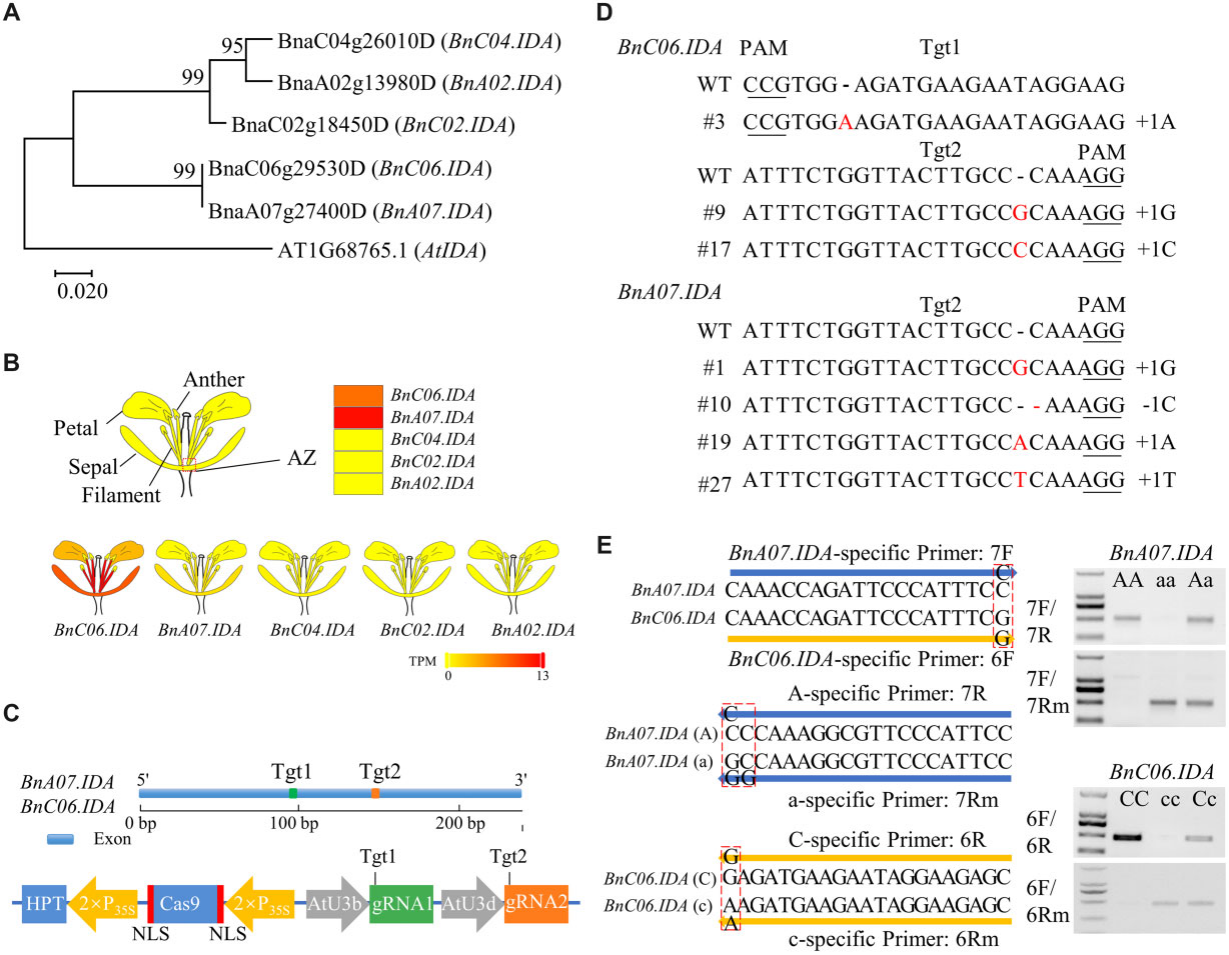
Generation of *bnida* mutants in *B. napus* by CRISPR/Cas9-mediated gene editing. A, Phylogenetic analysis of *IDA* homologs in *B. napus* and *Arabidopsis*. A neighbor joining tree is presented based on the deduced amino acid sequences. Bootstrap values (1,000 replicates) are shown at each branch as a percentage. A branch length scale bar indicates the number of amino acid residue substitutions per site. B, Expression patterns of *BnIDAs* in ﬂoral organs of *B. napus*. The expression of *BnIDAs* in the AZ of the flower at position 3 was determined by transcriptome sequencing of line J9712. The expression patterns in petals, sepals, and filaments were acquired from the BnTIR transcriptomic database (Liu et al., 2021). TPM, transcripts per million mapped reads. C, Schematics of the target sites (Tgt) of *BnA07.IDA* and *BnC06.IDA* and the Cas9/gRNA vector. NLS, nuclear location signal; HPT, hygromycin phosphotransferase. D, Sequences at the sgRNA target sites in the T_0_ generation according to TA cloning and Sanger sequencing. Dashes represent deletions; red letters represent insertions. The protospacer-adjacent motif (PAM) is underlined. E, Allele-specific markers were developed to discriminate the wild-type, heterozygous, and homozygous mutants for *BnA07.IDA* (AA, Aa, and aa, respectively) and *BnC06.IDA* (CC, Cc, and cc, respectively).

To investigate the function of *IDA* on ﬂoral organ abscission, *BnC06.IDA* and *BnA07.IDA* were knocked out using CRISPR/Cas9 genome editing. To that end, two guide RNAs (gRNAs), gRNA1 and gRNA2, were designed to specifically target two regions of the coding sequence (Tgt1 and Tgt2) of *BnC06.IDA* and *BnA07.IDA* (Figure 1C). The CRISPR/Cas9 construct contained two gRNAs driven by *Arabidopsis* U3b and U3d promoters, and a Cas9 gene driven by the 35S promoter (Figure 1C).

The construct was transformed into J9712 through *Agrobacterium*-mediated transformation, and a total of 30 independent positive transformants were obtained. Seven of the 30 (23.3%) T_0_ plants exhibited editing events at the designed target sites (Figure 1D). All mutant plants were single heterozygous mutants with single nucleotide deletions or insertions that led to translational frame shifts (Figure 1D). No editing events occurred at *BnC04.IDA*, *BnA02.IDA*, and *BnC02.IDA*.

Mutant plants were self-pollinated and the obtained T_1_ progeny contained single homozygous mutations in *bna07.ida* and *bnc06.ida*. However, ﬂoral abscission in *bnc06.ida* and *bna07.ida* single mutants was similar to wild-type plants (J9712) (Figure 2A), suggesting functional redundancy between the *BnIDA* homologs. To obtain *bnc06.ida bna07.ida* double mutants, we crossed single mutants of *bna07.ida* (line #10) and *bnc06.ida* (line #3). To discriminate wild-type, heterozygous, and homozygous mutants, allele-speciﬁc PCR markers for *BnA07.IDA* and *BnC06.IDA* were developed (Figure 1E and Supplemental Table S1). Marker-assisted selection and a comprehensive speed breeding system (Song et al., 2022) allowed us to successfully obtain F_2_ progeny with *bnc06.ida bna07.ida* double mutants within 6 months.

**Figure 2:**
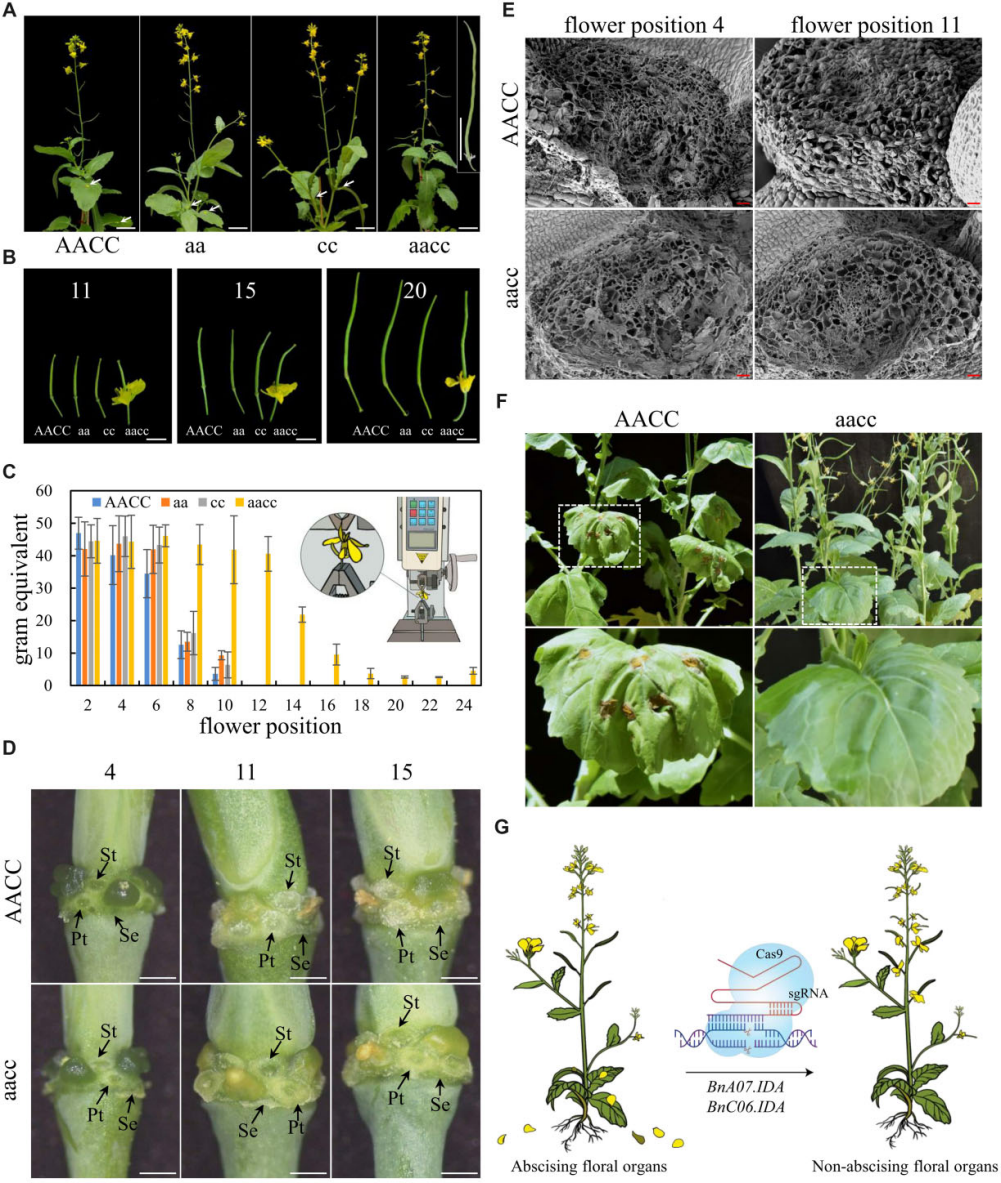
Phenotype of *bna07.ida* (aa), *bnc06.ida* (cc), and *bna07.ida bnc06.ida* (aacc) compared with the wild-type (AACC). A, The whole inflorescences of wild-type and *BnIDA* mutants. Red arrows indicate petals and stamens adhering to leaves or petioles after ﬂoral organ abscission. Flowers from *bna07.ida bnc06.ida* plants displayed an abscission-defective phenotype. Scale bar = 5 cm. The upper right photograph shows that flowers remained attached to mature siliques. B, Representative siliques from wild-type and *BnIDA* mutants at ﬂower positions 11, 15, and 20. Scale bar = 1 cm. Position 1 refers to the youngest flower with visible yellow petals at the top of the inflorescence. C, Petal breakstrength of flowers at different positions of the wild-type and *BnIDA* mutants. Error bars indicate standard deviations (*n* = 12) for each position. D and E, Morphology of floral AZ at different flower positions. D, Secretion of the white substance at petal (Pt), sepal (Se), and stamen (St) AZ after abscission in wild-type plants. Scale bar = 1 mm. E, Scanning electron microscopy of the petal AZ cells at positions 4 and 11. Scale bar = 30 μm. F, Assessment of the sensitivity of *bna07.ida bnc06.ida* mutant and wild-type plants to *S. sclerotiorum*. The enlarged images show magnified views of the red boxes above. G, Schematic diagram showing the generation of oilseed rape with non-abscising ﬂoral organs by CRISPR/Cas9.

Wild-type and mutant plants were subsequently grown in a climate chamber. The sepals, petals, and stamens abscised at flower positions 10–11 in wild-type and *bna07.ida bnc06.ida* plants (Figure 2, A and B). Strikingly, the double mutants displayed a ﬂoral abscission-defective phenotype in which floral organs remained attached to developing siliques, and dry and colorless senesced floral parts remained attached to mature siliques (Figure 2, A and B). In contrast, the detached petals and stamens in wild-type and single mutants of *bna07.ida* and *bnc06.ida* adhered to leaves or petioles (Figure 2A).

To quantify the extent of floral organ abscission, the mechanical force required to remove petals from flowers was measured with a high-precision tension meter (Figure 2C). The breakstrength of wild-type petals dramatically decreased at positions 8 and 10, and were zero after position 10 since the petals were already detached (Figure 2C). By contrast, mechanical force was required to remove the petals at all tested positions in the *bnc06.ida bna07.ida* plants (Figure 2C).

We further compared the morphological differences at the floral AZ between the wild-type and *bna07.ida bnc06.ida* plants. At position 4, floral organs were forcibly removed, forming a fracture plane with broken cells at the floral AZ in both genotypes (Figure 2, D and E). After abscission, beginning at position 11 of wild-type plants, the floral AZ was covered with a white substance rich in arabinogalactan (as described by Stenvik et al., 2006; Figure 2D), and the AZ cells were enlarged and rounded to form an abscission scar (Figure 2E). Instead of rounded cells and white substance, broken cells were found in the floral AZ of double mutant plants at position 11 (Figure 2, D and E), indicating incomplete dissolution of the middle lamella in the AZ cells. Apart from floral abscission, the majority of agronomic traits and fertility between *bna07.ida bnc06.ida* and wild-type plants were not substantially different (Supplemental Table S2 and Supplemental Figure S2).

To test sensitivity of the mutants to SSR, petals were inoculated with a mycelial suspension of *Sclerotinia*. In wild-type plants, inoculated petals were removed and adhered to leaves, while inoculated petals remained attached in the mutants. The majority of infected petals caused necrosis to the leaves of wild-type plants, whereas disease lesions were undetectable in the petals and leaves of the double mutants (Figure 2F).

In summary, our results demonstrate that *BnA07.IDA* and *BnC06.IDA* are functionally redundant and are involved in the regulation of ﬂoral abscission in OSR. We successfully generated OSR plants with abscission-defective ﬂoral organs by simultaneously inactivating two *BnIDA* genes using CRISPR/Cas9 (Figure 2G). The double mutant line, with its allele-speciﬁc markers, can be applied for breeding OSR cultivars with non-abscising ﬂoral organs that are anticipated to lengthen the ﬂowering period for ornamental varieties, and to avoid *Sclerotinia* infection via abscised petals and stamens.

## Supplemental data

The following materials are available in the online version of this article.

**Supplemental Figure S1.** Petals adhered to leaves and petioles after abscission, spreading SSR.

**Supplemental Figure S2.** Fertility performances of *bna07.ida bnc06.ida* and wild-type plants.

**Supplemental Table S1.** Sequences of the primers used in this study.

**Supplemental Table S2.** Agronomic traits of wild-type and *bna07.ida bnc06.ida* plants grown in the climate chamber.

**Supplemental Materials and Methods**.

## Supplementary Material

kiac364_Supplementary_Data

## Notes

J.W. and Y.W. conceived the study. J.W., H.L., S.R., P.L., X.L., L.L., and L.Z. performed the experiments. J.W. wrote the manuscript. Q.S. and C.L. revised the manuscript.

The author responsible for distribution of materials integral to the findings presented in this article in accordance with the policy described in the Instructions for Authors (https://academic.oup.com/plphys/pages/general-instructions) is: Youping Wang (wangyp@yzu.edu.cn).
