# Supplementary material for: Generating an oilseed rape mutant with non-abscising floral organs using CRISPR/Cas9 technology
Source: Plant Physiol. 2022 Aug 11;190(3):1562–5. doi: 10.1093/plphys/kiac364 (PMC9614459; doi:10.1093/plphys/kiac364)
Supplement: kiac364_Supplementary_Data [file kiac364_supplementary_data.zip › Supplemental data.pdf]

Supplemental Materials and methods

1. Plant material and growth conditions

B. napus line J9712 was kindly provided by Prof. Dr. Yongming Zhou (Huazhong Agricultural University, Wuhan, Hubei, China). All plants were grown in a climate chamber under a 22 h light /2 h dark photoperiod at 22 °C with a humidity of 65%.

2. RNA sequencing of the floral abscission zone (AZ)

RNA sequencing was performed to reveal the expression of the five *BnIDA* genes in the floral abscission zone (AZ) of J9712. Floral AZs from flower positions 3 to 6 were collected for RNA sequencing, where position 1 refers to the youngest flower with visible yellow petals at the top of the inflorescence. Harvested AZs were immediately frozen in liquid nitrogen and stored at -80 °C. Three biological replicates were performed, and fifty AZs from six plants were used for each biological replicate.

Total RNA was extracted using an RNAiso reagent kit (Vazyme, Nanjing, China) following the manufacturer's instructions. RNA samples were subjected to library construction using the VAHTS Universal V8 RNA-seq Library Prep Kit for MGI (Vazyme). All samples were sequenced with a DNBSEQ-T7 sequencer (MGI, Shenzhen, China) at the National Key Laboratory of Crop Genetic Improvement, Huazhong Agricultural University, generating 2×150 bp paired-end reads. RNA sequence analysis was performed as previously described (Wu et al., 2016). The original data set was deposited in the NCBI Sequence Read Archive (accession no. PRJNA848958).

3. Vector construction, plant transformation and mutant identification

Two sequence-specific sgRNAs were designed using the online software CRISPR-P 2.0 (http://crispr.hzau.edu.cn/cgi-bin/CRISPR2/CRISPR). A CRISPR/Cas9 construct containing two sgRNA cassettes was generated according to Ma et al. (2015) using the binary pYLCRISPR/Cas9 multiplex genome targeting vector, kindly provided by Prof. Dr. Yaoguang Liu at South China Agricultural University, Guangzhou, Guangdong, China.

The CRISPR/Cas9 construct was transformed into *Agrobacterium tumefaciens* (GV3101) by electroporation and further transformed into *B. napus* line J9712 with an *A. tumefaciens*-mediated hypocotyl method (Dai et al., 2020). Positive transformants harboring T-DNA insertions were identified by polymerase chain reaction (PCR) with specific primers (Supplemental Table S1). DNA fragments containing target sequences were amplified by PCR with gene-specific primers (Supplemental Table S1), and the editing events at the designed target sites were detected with TA cloning and Sanger sequencing.

4. Petal breakstrength measurement

The mechanical force required to remove petals (petal breakstrength) at different flower positions in wildtype and *BnIDA* mutant plants was determined with a high-precision digital tension meter (SH-2, SHSIWI, Shanghai, China). Two petals per position from six plants of each genotype were assayed.

5. Light microscopy and scanning electron microscopy

After floral organs (petals, sepals and stamens) were forcibly removed or had naturally abscised, morphology of the floral AZ was visualized and photographed with a Leica EZ4 stereomicroscope (Leica, Wetzlar, Germany).

For scanning electron microscopy (SEM) analysis, floral AZ sections collected from positions 4 and 11 were fixed in 2.5% glutaraldehyde (w/v) at 4 °C for up to 4 h. After washing with 0.1 M potassium phosphate buffer (pH 7.5) 3-4 times, the samples were dehydrated for 15 min in each concentration of ethanol in a series (30%, 50%, 70%, 80%, 90% and 100%). The samples were then dried with an automated critical point dryer (Leica EM CPD 300). After being sprayed with gold in an MC1000 ion sputtering apparatus (Hitachi, Tokyo, Japan), dried AZs were photographed by a field emission scanning electron microscope system (GeminiSEM 300, Carl Zeiss, Jena, Germany).

6. Evaluation of plant sensitivity to *S. sclerotiorum*

S. sclerotiorum isolate SS-1 was cultured on potato dextrose agar (PDA, Becton, Dickinson and Company, Franklin Lakes, NJ, USA) at 23 °C in the dark. Six mycelial agar plugs (5-mm in diameter) were excised from the actively growing margin of a 2-day-old colony. The mycelial agar plugs were further cultured for three days in a 250-mL flask containing 200 mL potato dextrose broth (PDB; Becton, Dickinson and Company, New Jersey, USA) at 23 °C on a platform shaker at 150 rpm min^{-1}. Cultured mycelial balls were harvested and washed twice with sterile distilled water, transferred into 100 mL PDB and pulverized by high-speed homogenization (ULTRA-TURRAX T18 digital, IKA, Staufen, Germany) at 10,000 r min^{-1} for 10 min. Finally, the mycelial suspension was filtered through degreased gauze and adjusted to a concentration of 10^5 fragments mL^{-1} with PDB with a hemocytometer. Mycelial suspension (10 μL) was deposited onto each petal from positions 7 to 10 using a micropipette. To mimic petal abscission in a natural situation, inoculated wildtype petals were removed and adhered to wildtype leaves, while inoculated mutant petals remained attached in the mutants. The degree of SSR infection of plants was evaluated 36 h post-inoculation.

Dai C, Li Y, Li L, Du Z, Lin S, Tian X, Li S, Yang B, Yao W, Wang J, et al. (2020) An efficient *Agrobacterium*-mediated transformation method using hypocotyl as explants for *Brassica napus*. Mol Breeding **40**: 96

Ma X, Zhang Q, Zhu Q, Liu W, Chen Y, Qiu R, Wang B, Yang Z, Li H, Lin Y, et al. (2015) A robust CRISPR/Cas9 system for convenient, high-efficiency multiplex genome editing in monocot and dicot plants. Mol Plant **8**: 1274–1284

Wu J, Zhao Q, Yang Q, Liu H, Li Q, Yi X, Cheng Y, Guo L, Fan C, Zhou Y (2016) Comparative transcriptomic analysis uncovers the complex genetic network for resistance to *Sclerotinia sclerotiorum* in *Brassica napus*. Sci Rep-UK **6**: 19007

Supplemental Table S1. Sequences of the primers used in this study.

Primer name	Sequence (5'-3')	Usage
IDA 1F	CATCAAATCCACACACACGC	Cloning of *BnC06.IDA* and *BnA07.IDA*
IDA 1R	CGGATGATGTGTGATGCTGGA	
IDA 2F	ACCATTCCACTCTTTTCCCCAT	Cloning of *BnA02.IDA*
IDA 2R	AAGCGGCTGATGGGTGATGC	
IDA 3F	CCATTCCACTCTTTTCGCCA	Cloning of *BnC04.IDA*
IDA 3R	AAGCGGCTGAAGGGTGATGC	
IDA 4F	TTCCCCACATTTTCGCCACA	Cloning of *BnC02.IDA*
IDA 2R	AAGCGGCTGATGGGTGATGC	
IDA 6F	CAAACCAGATTCCCATTTCG	Allele-specific marker for *BnC06.IDA*
IDA 6R	GCTCTTCCTATTCTTCATCTC	
IDA 6F	CAAACCAGATTCCCATTTCG	Allele-specific marker for *bnc06.ida*
IDA 6Rm	GCTCTTCCTATTCTTCATCTT	
IDA 7F	CAAACCAGATTCCCATTTCC	Allele-specific marker for *BnA07.IDA*
IDA 7R	GGAATGGGAACGCCTTTGGG	
IDA 7F	CAAACCAGATTCCCATTTCC	Allele-specific marker for *bna07.ida*
IDA 7Rm	GGAATGGGAACGCCTTTGCC	
SP-DL	CCGGTCAACATGTGGAGCACGAC	Identification of positive transgenic plants
Tgt-1R	CAATGAGGATGAGAGTCAA	
IDA 6F	CAAACCAGATTCCCATTTCG	PCR amplification of target fragment of *BnC06.IDA*
IDA 1R	CGGATGATGTGTGATGCTGGA	
IDA 7F	CAAACCAGATTCCCATTTCC	PCR amplification of target fragment of *BnA07.IDA*
IDA 1R	CGGATGATGTGTGATGCTGGA	

Supplemental Table S2. Agronomic traits of wild-type and *bna07.ida bnc06.ida* plants grown in the climate chamber.

Line	Plant height （cm）	Branch initiation height (cm)	First effective branch number	Silique number per plant	Silique lengh (cm)	Silique seed number	Thousand-seed weight （g）
J9712	107.36 ± 8.32	41.55 ± 6.35	3.91 ± 0.94	57.36 ± 16.15	11.15 ± 2.08	24.38 ± 0.34	4.08 ± 0.13
bna07.ida bnc06.ida	100.17 ± 8.97	36.50± 8.10	4.00 ± 1.28	69.83 ± 24.33	11.48 ± 2.18	24.62 ± 0.14	4.05 ± 0.11

n = 15, mean ± SD.

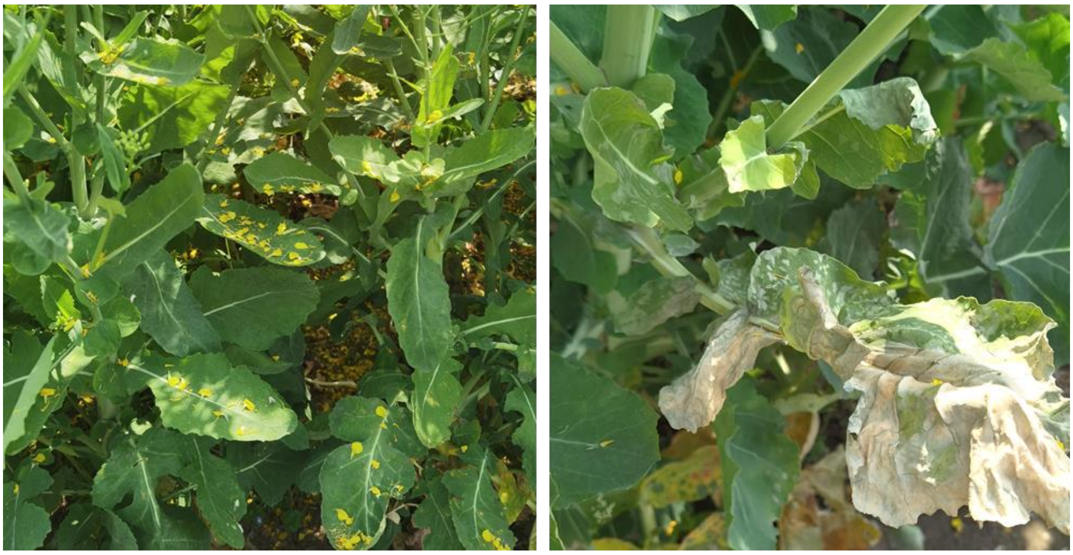

Supplemental Figure S1. Petals adhered to leaves and petioles after abscission, spreading Sclerotinia stem rot.

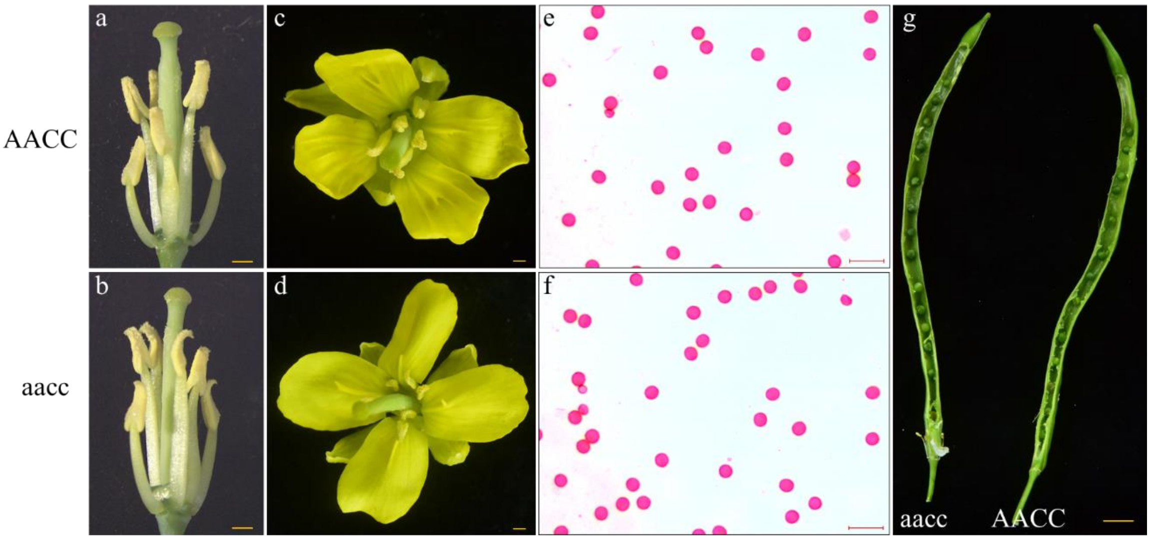

Supplemental Figure S2. Fertility performances of *bna07.ida bnc06.ida* and wild-type plants. (a, b) petal-removed flowers; (c, d) flowers; (e, f) morphology of pollen grains stained with 1% aceto-carmine staining solution; (g) siliques under artificial pollination. Scale bars, 1 mm in (a) to (d), 100 µm in (e) and (f), and 1 cm in (g).
