# Supplementary material for: Generating an oilseed rape mutant with non-abscising floral organs using CRISPR/Cas9 technology
Source: Plant Physiol. 2022 Aug 11;190(3):1562–5. doi: 10.1093/plphys/kiac364 (PMC9614459; doi:10.1093/plphys/kiac364)
Supplement: kiac364_Supplementary_Data [file kiac364_supplementary_data.zip › Table S2.pdf]

Supplemental Table S2. Agronomic traits of the wildtype and *bna07.ida bnc06.ida* plants under controlled-climate chamber conditions.

**Line**	**Plant height （cm）**	**Branch initiation height (cm)**	**First effective branch number**	**Silique number per plant**	**Silique lengh (cm)**	**Silique seed number**	**Thousand-seed weight（g）**
J9712	107.36 ± 8.32	41.55 ± 6.35	3.91 ± 0.94	57.36 ± 16.15	11.15 ± 2.08	24.38 ± 0.34	4.08 ± 0.13
bna07.ida bnc06.ida	100.17 ± 8.97	36.50± 8.10	4.00 ± 1.28	69.83 ± 24.33	11.48 ± 2.18	24.62 ± 0.14	4.05 ± 0.11
