# Supplementary figures and images for: Generating an oilseed rape mutant with non-abscising floral organs using CRISPR/Cas9 technology
Source: Plant Physiol. 2022 Aug 11;190(3):1562–5. doi: 10.1093/plphys/kiac364 (PMC9614459; doi:10.1093/plphys/kiac364)

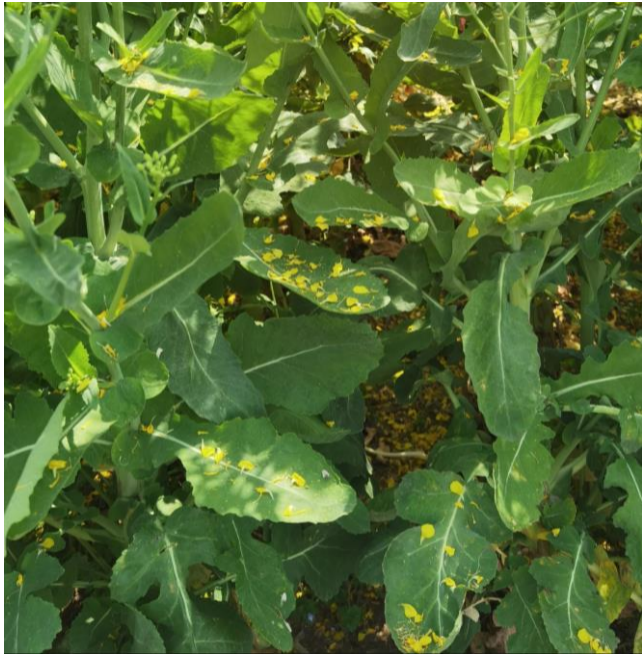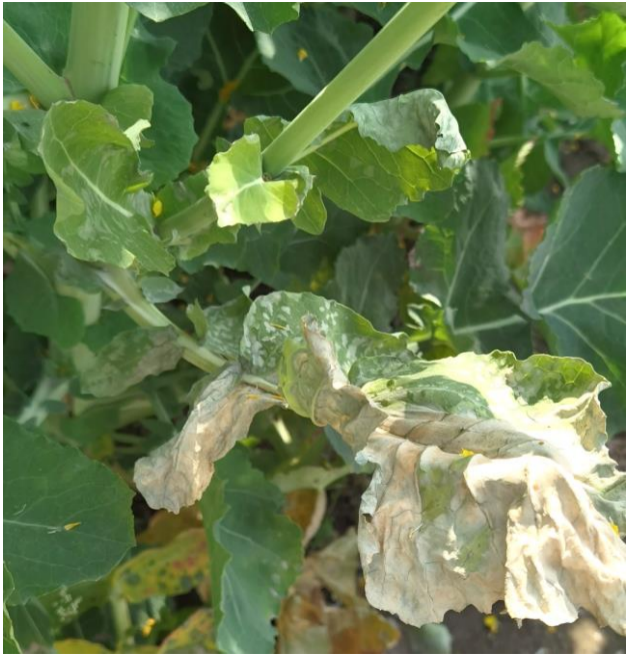

Supplement: kiac364_Supplementary_Data [file kiac364_supplementary_data.zip › FigS1Revise.pdf]

AACC

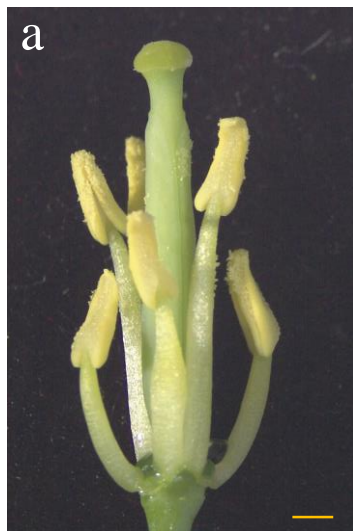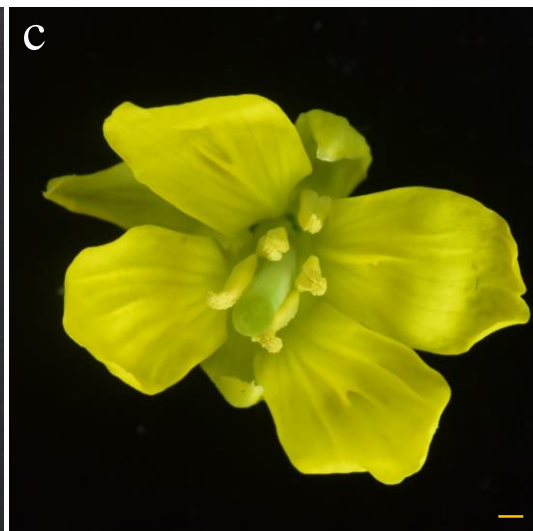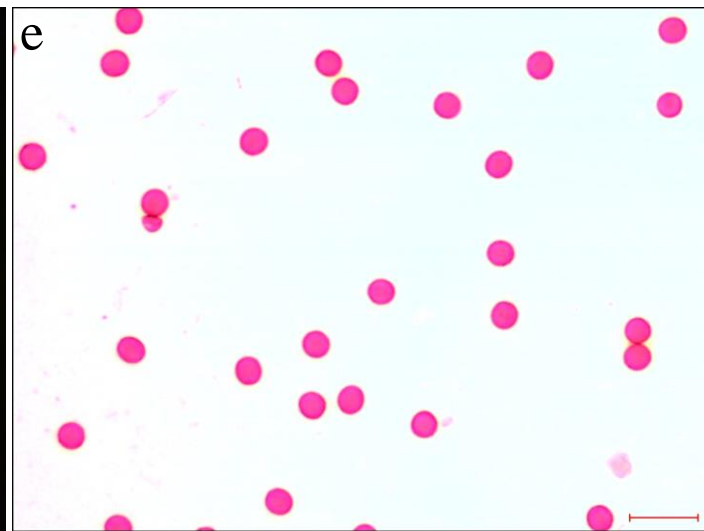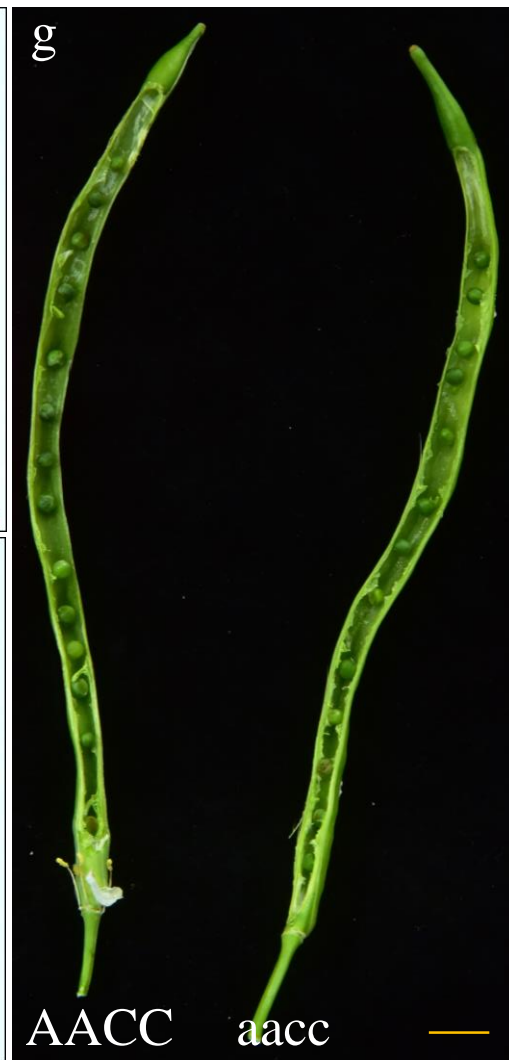

aacc

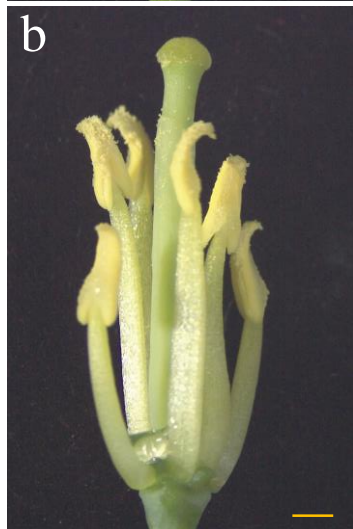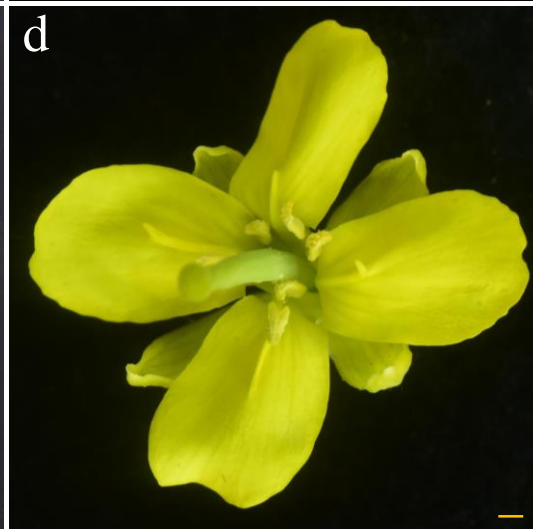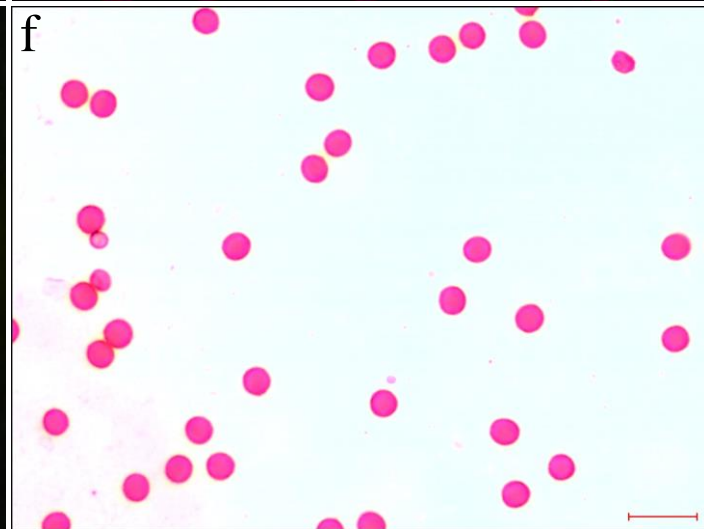

AACC

aacc

Supplement: kiac364_Supplementary_Data [file kiac364_supplementary_data.zip › FigS2Revise.pdf]
